# Corticotropin-releasing factor-related peptides, serotonergic systems, and emotional behavior

**DOI:** 10.3389/fnins.2013.00169

**Published:** 2013-09-20

**Authors:** James H. Fox, Christopher A. Lowry

**Affiliations:** Behavioral Neuroendocrinology Laboratory, Department of Integrative Physiology and Center for Neuroscience, University of Colorado BoulderBoulder, CO, USA

**Keywords:** anxiety, corticotropin-releasing factor, dorsal raphe nucleus, emotional behavior, serotonin

## Abstract

Corticotropin-releasing factor (CRF) is a 41-amino acid neuropeptide that is involved in stress-related physiology and behavior, including control of the hypothalamic-pituitary-adrenal (HPA) axis. Members of the CRF family of neuropeptides, including urocortin 1 (UCN 1), UCN 2, and UCN 3, bind to the G protein-coupled receptors, CRF type 1 (CRF_1_) and CRF_2_ receptors. In addition, CRF binding protein (CRFBP) binds both CRF and UCN 1 and can modulate their activities. There are multiple mechanisms through which CRF-related peptides may influence emotional behavior, one of which is through altering the activity of brainstem neuromodulatory systems, including serotonergic systems. CRF and CRF-related peptides act within the dorsal raphe nucleus (DR), the major source for serotonin (5-HT) in the brain, to alter the neuronal activity of specific subsets of serotonergic neurons and to influence stress-related behavior. CRF-containing axonal fibers innervate the DR in a topographically organized manner, which may contribute to the ability of CRF to alter the activity of specific subsets of serotonergic neurons. CRF and CRF-related peptides can either increase or decrease serotonergic neuronal firing rates and serotonin release, depending on their concentrations and on the specific CRF receptor subtype(s) involved. This review aims to describe the interactions between CRF-related peptides and serotonergic systems, the consequences for stress-related behavior, and implications for vulnerability to anxiety and affective disorders.

## Introduction

Corticotropin-releasing factor (CRF) is a 41-amino acid neuropeptide that is involved in stress-related physiology and behavior, including control of the hypothalamic-pituitary-adrenal (HPA) axis (Vale et al., 1981, 1983). CRF has been implicated in the etiology and pathophysiology of stress-related disorders such as anxiety and affective disorders (Dunn and Berridge, 1990; Binder and Nemeroff, 2010). One of the ways in which CRF may play a role in the etiology and pathophysiology of anxiety and affective disorders is through modulation of brainstem neuromodulatory systems such as serotonergic systems. Serotonin (5-hydroxytryptamine; 5-HT) has long been implicated in control of emotional behavior as well as anxiety and affective disorders (Ressler and Nemeroff, 2000). Consequently, understanding the interactions between CRF, CRF-related neuropeptides, and serotonergic systems is likely to lead to advances in understanding the biological basis of anxiety and affective disorders. This review aims to describe the interactions among CRF, CRF-related neuropeptides, and serotonergic systems and the importance of these interactions in modulating emotional behaviors involved in anxiety and affective disorders.

## CRF family of peptides

The CRF family of neuropeptides includes CRF as well as the urocortins (UCN), UCN 1, UCN 2, and UCN 3, structurally related peptides that have been discovered more recently (Vaughan et al., 1995; Donaldson et al., 1996; Zhao et al., 1998; Lewis et al., 2001; Reyes et al., 2001; Lovejoy and Jahan, 2006; Fekete and Zorrilla, 2007). UCN 1 is a 40-amino acid peptide while both UCN 2 and 3 are 38-amino acid peptides. The UCN's, like CRF, have been implicated in stress-related physiology and behavior, including modulation of the HPA axis (Vaughan et al., 1995; Reul and Holsboer, 2002). There are two receptors that CRF and the UCN's bind to with high affinity, which are designated as CRF_1_ (Perrin et al., 1993) and CRF_2_ receptors (Lovenberg et al., 1995). They are both G protein-coupled receptors belonging to the B1 sub-family of G-coupled receptors and couple to both *G*_*s*_ and *G*_*q*_ (Perrin et al., 2006) with varying affinities for the neuropeptides in the CRF family. CRF itself has a greater affinity for CRF_1_ receptors while UCN 1 binds with high affinity to both receptors and UCN 2 and UCN 3 both preferentially bind to CRF_2_ receptors (Vaughan et al., 1995; Lewis et al., 2001; Reyes et al., 2001). Several splice variants for both receptor subtypes have also been reported and the structural and functional properties of these splice variants have been reviewed previously (Dautzenberg et al., 2001). Finally, the CRF binding protein (CRFBP) shows high affinity for both CRF and UCN 1 but has little affinity for UCN 2 or 3 (Lewis et al., 2001).

### Distribution of CRF containing neurons in neural circuits controlling emotional behavior

Corticotropin-releasing factor-containing neurons are widely distributed throughout both the rat and mouse brains, with several areas differing in expression levels, based on patterns of immunohistochemical staining in the two species (Wang et al., 2011). Given the wide distribution of CRF-containing neurons within the central nervous system, the idea that CRF works as a neuromodulator has received considerable attention in the past few decades. The main focus of this review is the role of CRF and CRF-related neuropeptides in stress-related emotional behavior, and therefore we focus on the distribution of these neuropeptides in neural circuits implicated in control of stress-related emotional behavior. A full consideration of the distribution of CRF and CRF-related neuropeptides can be found in previous reviews focusing on the chemical neuroanatomy (Swanson et al., 1983; Sakanaka et al., 1987; Kozicz, 2007).

A major source for CRF in the brain is the paraventricular nucleus of the hypothalamus (PVN) (Sakanaka et al., 1987). CRF synthesized in the PVN, via projections to the median eminence, plays a primary role in control of the HPA axis. However, several extrahypothalamic brain regions involved in control of emotional behavior have CRF-containing neurons. In particular, both the central nucleus of the amygdala (CE) and the bed nucleus of the stria terminalis (BNST) contain CRF-immunoreactive neurons with extensive projections to brainstem structures controlling emotional behavior (Gray, 1993; Wang et al., 2011). Other regions with CRF expressing neurons that are involved in control of emotional behavior include the hippocampus, subiculum, lateral septum, and periaqueductal gray (Sakanaka et al., 1987; Calandreau et al., 2007). The localization of CRF in brain regions involved in control of emotional behavior implicated CRF as an important neuromodulator, in addition to an important neurohormonal function (Gray, 1993).

### Distribution of UCN 1, 2, and 3 containing neurons

The UCN's are expressed in discrete regions within the brain. The non-preganglionic Edinger-Westphal nucleus has a large number of UCN 1 neurons (Kozicz et al., 1998). Additionally, the lateral superior olivary and supraoptic nuclei also have been shown to have mRNA and immunoreactivity for UCN 1 (Bittencourt et al., 1999; Lewis et al., 2001). UCN 2 is mainly localized in subcortical structures including the locus coeruleus (Reyes et al., 2001). UCN 3 is also localized to discrete areas of the brain including an area encircling the columns of the fornix in the rostral hypothalamus, the posterior portion of the BNST and an area dorsolateral to the caudal portion of the dorsomedial hypothalamic nucleus (Kuperman et al., 2010). Another grouping of UCN 3 neurons is located in the anterodorsal part of the medial amygdaloid nucleus (Lewis et al., 2001; Li et al., 2002).

### Distribution of CRF receptors in emotion-related brain regions

The distribution of CRF_1_ and CRF_2_ receptors within rodent brain has been well-described with CRF_1_ receptors being more widely distributed while CRF_2_ receptors are more restricted to subcortical areas (Potter et al., 1994; Chalmers et al., 1995; Van Pett et al., 2000). The hippocampus contains both CRF receptors as does the periaqueductal gray (Van Pett et al., 2000). The amygdala expresses both receptor subtypes with low levels of only CRF 1 receptors in the CE (Van Pett et al., 2000). All portions of the BNST have been shown to have CRF_1_ receptors while the posterior portion of the BNST also has CRF_2_ receptors (Van Pett et al., 2000). Importantly for this review, the raphe nuclei including the DR and median raphe nucleus (MnR) both have CRF_1_ and CRF_2_ receptors with the DR having higher levels of CRF_2_ and the MnR having about equal amounts of both receptors (Van Pett et al., 2000; Day et al., 2004).

## The functional subsets of 5-HT neurons based on functional neuroanatomy and afferent and efferent connections

In order to discuss the possibility that CRF and CRF-related peptides control functional subsets of serotonergic neurons involved in control of emotional behavior, it is first useful to consider the evidence for a topographical and functional organization of the midbrain raphe complex. The midbrain raphe complex includes serotonergic systems located within the DR, the median raphe nucleus, caudal linear nucleus, pontomesencephalic reticular formation, supralemniscal cell group, and interpeduncular nucleus (Hale et al., 2012) Here we will focus on the organization of the DR. The DR is topographically organized and can be divided into subregions making up the rostral, dorsal, ventral, ventrolateral, interfascicular, and caudal portions. It is beyond the scope of this review to describe in detail the topography but we will, in brief, describe the major subdivisions here and refer the reader to previous reviews for a thorough review of the DR serotonergic system and its topography (Lowry, 2002; Lowry et al., 2005, 2008; Hale and Lowry, 2011; Hale et al., 2012).

### The rostral DR

The rostral portion of the DR, which is located from approximately −7.04 to −7.30 mm from bregma in the rat brain (Paxinos and Watson, 1998), receives projections from cingulate, orbital and infralimbic cortices, as well as a small number of projections from the CE, BNST, and substantia inominata and larger numbers from the paraventricular and other hypothalamic nuclei (Peyron et al., 1997). In turn, the rostral DR projects to the caudate putamen with collaterals to the substantia nigra, and also projects to the subthalamic nucleus and substantia inominata (Steinbusch, 1981; Imai et al., 1986; Canteras et al., 1990; Grove, 1998). Data show that 6 weeks of voluntary wheel running increases 5-HT_1A_ receptor mRNA in the rostral and mid-rostrocaudal DR as well as decreases 5-HT_1B_ receptor and 5-HT transporter mRNA (Greenwood et al., 2003, 2005). Voluntary wheel running is also associated with a protective effect against the behavioral deficits associated with uncontrollable tail shock such as exaggerated freezing in a shuttle box (Greenwood et al., 2003, 2005). These data show that the rostral DR is connected with emotion-related brain regions and that altered emotional behavior is associated with serotonergic changes in this brain region.

### The dorsal part of the DR

**Figure 1:**
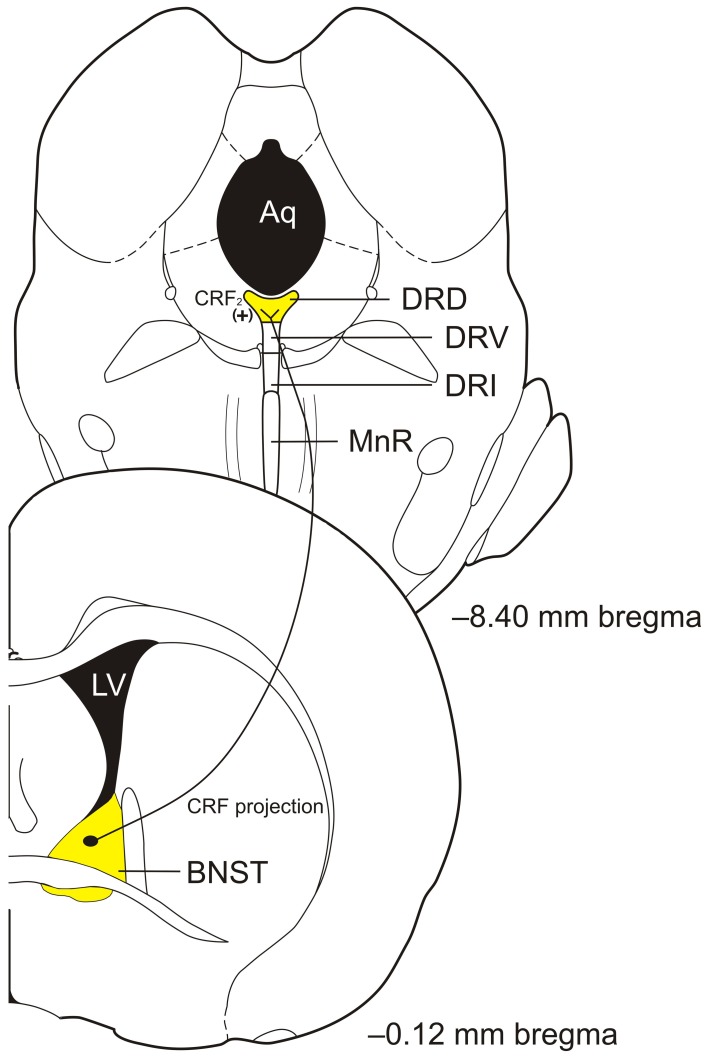
**Diagram of proposed bed nucleus of the stria terminalis (BNST) corticotropin-releasing factor (CRF) projections to the caudal portion of the dorsal part of the dorsal raphe nucleus (DRD) to activate CRF_2_ receptors to modulate serotonin (5-hydroxytryptamine; 5-HT) output in control of anxiety-like behaviors.** Abbreviations: Aq, aqueduct; CRF, corticotropin-releasing factor; DRD, dorsal part of the dorsal raphe nucleus; DRI, interfascicular part of the dorsal raphe nucleus; DRV, ventral part of the dorsal raphe nucleus; LV, lateral ventricle; MnR, median raphe nucleus; (+), excitation. Coronal section templates reproduced from Paxinos and Watson (1998), The Rat Brain in Stereotaxic Coordinates, 4th Edition. San Diego: Academic Press: 1998 with permission from Elsevier.

The DRD is located from approximately −7.30 to −8.30 mm from bregma in the rat brain (Paxinos and Watson, 1998). The DRD receives projections from areas associated with the control of emotional behaviors including the lateral and ventral orbitofrontal and infralimbic cortices, CE, BNST, and the dorsal, dorsomedial, lateral, and posterior hypothalamic nuclei (Peyron et al., 1997). Further, the DRD projects to areas associated with control of emotional behaviors including the CE, BLA, BNST, nucleus accumbens (Acb), medial prefrontal cortex (mPFC), and dorsal hypothalamus (Van Bockstaele et al., 1993; Commons et al., 2003; Hale et al., 2008). In addition, the DRD sends a number of collateral projections to functionally related forebrain targets involved in emotional behavior. Anxiety related stimuli such as multiple classes of anxiogenic drugs including UCN 2, or anxiety producing situations including exposure to an open-field test arena, or social defeat, lead to increased activation of DRD serotonergic neurons as measured by c-Fos immunoreactivity (Abrams et al., 2005; Gardner et al., 2005; Bouwknecht et al., 2007; Hale et al., 2008, 2010; Paul et al., 2011). Lastly, chronic corticosterone in the drinking water, which increases anxiety-like behavior in a social interaction task, open field task, and elevated plus maze, increases tryptophan hydroxylase 2 (TPH) mRNA in the DRD (Donner et al., 2012b). The DRD is connected with emotion-related brain regions including the BNST (see Figure 1) and activation by anxiogenic stimuli show that it may be an important region involved in the control of emotion-related behavioral output.

### The ventral part of the DR

The DRV is located from approximately −7.30 to −8.30 mm from bregma in the rat brain (Paxinos and Watson, 1998). The DRV receives projections from the cingulate and lateral orbital cortices, CE, and dorsomedial hypothalamic nucleus, with less dense projections from other amygdaloid nuclei and cortex (Peyron et al., 1997). The DRV in turn projects to sensorimotor, ventrolateral orbital, frontal, motor, and visual cortices and the caudate putamen (Steinbusch et al., 1980; Steinbusch, 1981; Waterhouse et al., 1986; Kazakov et al., 1993). Because of the projections to frontal, visual, and motor cortex, it is likely that this region of the DR is involved in directed behaviors that may or may not have an emotional content. Further research of DRV serotonergic neurons is needed for a better understanding of their functional properties.

### The ventrolateral part of the DR and ventrolateral periaqueductal gray

The DRVL/VLPAG is located lateral to the DRD and occurs approximately from −7.64 to −8.54 mm from bregma in the rat brain (Paxinos and Watson, 1998). The DRVL/VLPAG receives projections from a number of brain regions involved in autonomic and emotional control. These include projections from the amygdala, with heavy innervation by the CE and moderate innervation by the dorsolateral medial amygdala with additional projections from the ventromedial prefrontal cortex, hypothalamus, and the retina (Hurley et al., 1991; Shen and Semba, 1994; Lee et al., 2003, 2007). The DRVL/VLPAG gives rise to projections involved in visual function including the superior colliculus and lateral geniculate nucleus (O'Hearn and Molliver, 1984; Waterhouse et al., 1993). Further, the DRVL/VLPAG also projects to the hypothalamus, medulla, PAG, and subcortical somatosensory regions, and data also suggest that DRVL/VLPAG serotonergic neurons control, via multisynaptic connections, presympathomotor neurons in the spinal cord (Beitz, 1982; Stezhka and Lovick, 1997; Ljubic-Thibal et al., 1999; Underwood et al., 1999; Kirifides et al., 2001; Bago et al., 2002; Kerman et al., 2006). The DRVL/VLPAG connections suggest that this region is important in control over panic-like and fight-or-flight behaviors. Consistent with this hypothesis, data show that panic-inducing stimuli such as hypercapnia or sodium lactate activate DRVL/VLPAG serotonergic neurons, but not in rats that are made panic prone, suggesting that the DRVL/VLPAG may inhibit panic in normal rats (Johnson et al., 2005, 2008).

**Figure 2:**
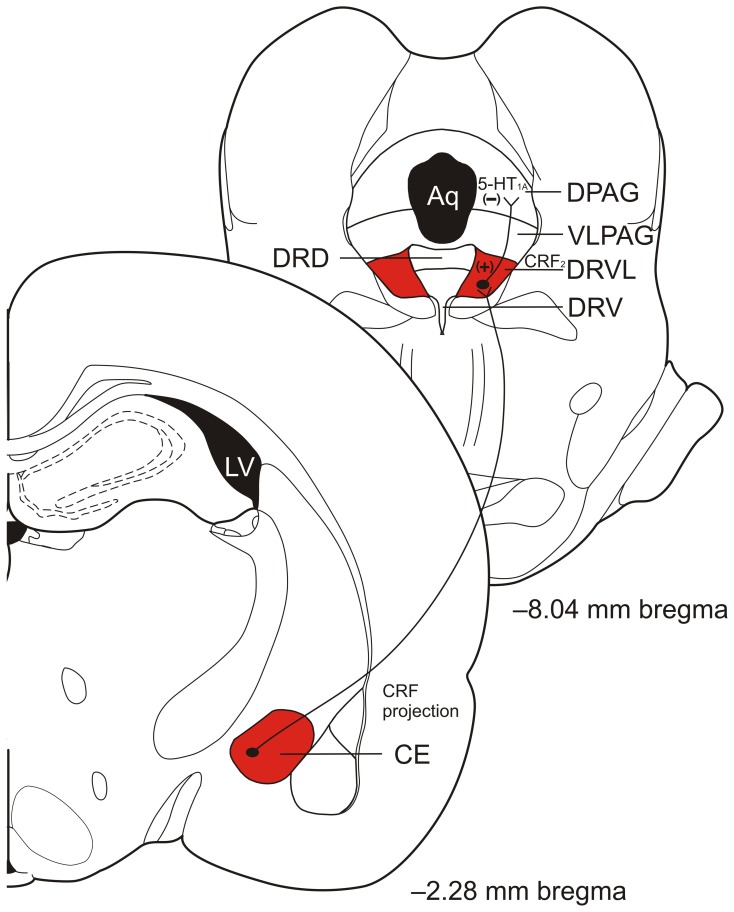
**Diagram of proposed central nucleus of the amygdala (CE) corticotropin-releasing factor (CRF) projections to the ventrolateral part of the dorsal raphe nucleus (DRVL) and DRVL serotonergic projections to the dorsal periaqueductal gray (DPAG) involved in panic inhibition during fear expression, such as freezing behavior.** Excitatory projections from the CE excite serotonergic neurons in the DRVL that in turn release serotonin (5-hydroxytryptamine; 5-HT) in the DPAG to act on inhibitory 5-HT_1A_ receptors to inhibit panic. Abbreviations: Aq, aqueduct; CE, central nucleus of the amygdala; CRF, corticotropin- releasing factor; DPAG, dorsal periaqueductal gray; DRD, dorsal part of the dorsal raphe nucleus; DRV, ventral part of the dorsal raphe nucleus; DRVL, ventrolateral part of the dorsal raphe nucleus; LV, lateral ventricle; VLPAG, ventrolateral periaqueductal gray; (+), excitation; (−), inhibition. Coronal section templates reproduced from Paxinos and Watson (1998), The Rat Brain in Stereotaxic Coordinates, 4th Edition. San Diego: Academic Press: 1998 with permission from Elsevier.

Recent data suggest that the DRVL/VLPAG may be an important component in the interdependence of fear- and panic-like responses. Data show that when a rat is fear conditioned and experiencing CE-mediated fear it is less likely to exhibit panic-like behaviors when given dorsal PAG electrical stimulation (Magierek et al., 2003). Clinical evidence supporting the hypothesis that fear may inhibit panic-like responses comes from a recent study in which 3 people with selective lesions of the amygdala were unable to experience normal fear but experienced panic when given CO_2_ inhalation (Feinstein et al., 2013). We propose that the CE, when activated, may serve to selectively inhibit panic through connections with DRVL/VLPAG serotonergic neurons (see Figure 2 for the proposed circuit). People with bilateral amygdala lesions are unable to inhibit CO_2_-induced panic responses while healthy controls generally do not experience panic when given CO_2_ (Goetz et al., 2001). The data thus suggest an important role of serotonergic neurons in the DRVL/VLPAG for control of emotional behaviors including fear and panic.

### The caudal portion of the DR

That most caudal subdivision of the DR (DRC) is located from approximately −8.30 to −9.30 mm bregma in the rat brain (Paxinos and Watson, 1998). The DRC receives a number of projections including from the mPFC, the preoptic area, arcuate nucleus, and perifornical and lateral hypothalamic areas, lateral habenula, and substantia nigra with more sparse afferents from both the CE and BNST (Lee et al., 2003). In turn, the DRC projects to brain regions involved in control of emotional behavior including the LC, amygdala, paraventricular nucleus of the thalamus and ventral hippocampus (Imai et al., 1986; Krout et al., 2002). Furthermore, the DRC has been shown to be involved in the behavioral deficits seen with inescapable stress and is activated following social defeat, administration of anxiogenic drugs, and administration of ligands including CRF and UCN 2 suggesting it, along with other regions of the DR, plays in an important role in the control of emotional behavior (Hammack et al., 2002, 2003a; Abrams et al., 2005; Gardner et al., 2005).

### The interfascicular part of the DR

The interfascicular part of the DR (DRI) is located approximately between −8.18 and −8.80 mm from bregma in the rat brain (Paxinos and Watson, 1998). Although not well-researched, studies have shown projections from the LC, median preoptic area, and the lateral parabrachial nucleus to the DRI (Saper and Loewy, 1980; Holstege, 1995; Lee et al., 2003; Kim et al., 2004). The DRI has, however, been shown to project to a number of regions. The DRI has projections to both dorsal and ventral hippocampus, medial septum, entorhinal, dorsolateral prefrontal, medial orbital, and anterior cingulate cortex and mediodorsal thalamus (Azmitia and Segal, 1978; Kohler and Steinbusch, 1982; Kohler et al., 1982; Porrino and Goldman-Rakic, 1982; Krout et al., 2002). The DRI, along with the DRVL/VLPAG, has been shown to be activated by a number of peripheral sensory stimuli, including peripheral injection of heat-killed *Mycobacterium vaccae* or lipopolysaccharide (LPS), and exposure to warm and cold temperature (Hollis et al., 2006; Hale et al., 2011; Kelly et al., 2011). Activation of DRI serotonergic neurons is associated with antidepressant-like behavioral responses (Lowry et al., 2007). This then suggests, consistent with its pattern of efferents that this area of the DR may also be important in controlling certain emotional behaviors.

### Distribution of 5-HT receptors in emotion related brain regions

There are at least 14 different 5-HT receptors that have been identified, all of which have been thoroughly reviewed previously (Hoyer et al., 1994, 2002; Barnes and Sharp, 1999; Smythies, 2005; Hannon and Hoyer, 2008). The receptors are divided into 7 families (1–7), and all, except the 5-HT_3_ receptors, are G protein-coupled metabotrobic receptors whereas the 5-HT_3_ receptor is a ligand-gated ion channel (Barnes and Sharp, 1999). Specifically, the 5-HT_1_ and 5-HT_5_ receptors are *G*_*i/o*_ coupled, 5-HT_2_ receptors are *G*_*q*/11_ coupled, and lastly, 5-HT_4_, 5-HT_6_, and 5-HT_7_ receptors are *G*_*s*_ coupled (Hannon and Hoyer, 2008). As well, 5-HT receptors are located both pre and post-synaptically, can be inhibitory or excitatory, and can be located on both γ-aminobutyric acid (GABA)ergic and glutamatergic neurons leading to a highly intricate and complex system within the brain (Rainnie, 1999; Guo and Rainnie, 2010).

Serotonin receptors are located in the amygdala and the BNST, which are thought to be important regions for fear and anxiety-behaviors. In addition, serotonin receptors have been implicated in playing a role in emotion-related behaviors in the hippocampus and the mPFC. Although all 5-HT receptors have been identified within the amygdala, particular attention has been paid to the 5-HT_1A_ and 5-HT_2C_ receptors (Park and Williams, 2012; Asan et al., 2013). Specifically, the 5-HT_1A_ receptors in the CE have been shown to be involved in the reduction of anxiety-like behaviors while 5-HT_2C_ receptors are associated with an increase in anxiety-like behaviors (Li et al., 2012). The hippocampus plays an important role in conditioned fear and 5-HT receptors appear to play an integral role (Eriksson et al., 2012). In the hippocampus, 5-HT_1A_ receptor activation appears to inhibit emotion-related behavior associated with fear conditioning (Stiedl et al., 2000). However, 5-HT_7_ receptor activation appears to enhance emotion-related behavior, especially when the 5-HT_1A_ receptors are blocked (Eriksson et al., 2012), while a lack of 5-HT_7_ receptors impairs fear learning (Roberts et al., 2004). Further, evidence suggests a role for activating 5-HT_2*A/C*_ receptors in the hippocampus in increasing GABA release (Shen and Andrade, 1998) while hippocampal 5-HT_2C_ activation has been associated with an increase in anxiety-like behaviors (Alves et al., 2004). Additionally, 5-HT_1A_, 5-HT_1*B*,_ 5-HT_2A_, 5-HT_2C_, and 5-HT_7_ receptors have been implicated in playing a role in emotional behaviors in the BNST, with the 5-HT_1_ receptor activation linked to reduced anxiety and the others linked to increased anxiety (Levita et al., 2004; Guo et al., 2009; Hammack et al., 2009; Guo and Rainnie, 2010). Lastly, the mPFC has been shown to be an important area involved in emotion-related behaviors and partially controlled by 5-HT (Amat et al., 2005). While 5-HT_2C_ receptors are located in this region, they have yet to be implicated in emotion-related behaviors outside of drug seeking (Pentkowski et al., 2010). However, 5-HT_1A_ receptors in the mPFC appear to play an important role in regulating 5-HT release from the DR such that stressful environments produce an increase in 5-HT release, which in turn appears to enact a negative feedback loop that turns off 5-HT release in the mPFC through glutamatergic and GABAergic neurons (Altieri et al., 2012). While both inhibitory and excitatory receptors are located in these brain regions, it is likely they work in concert depending on the context of the environment. For example, anxiety and fear are restrained unless the situation dictates the appropriateness of these emotions or in terms of psychopathology, these systems are no longer in concert to restrain anxiety and fear in inappropriate situations (Hammack et al., 2009).

### Distribution of CRF and CRF receptors within the dorsal raphe nucleus

As discussed briefly above, one mechanism through which CRF and the UCN's can influence emotional behavior is through actions on brainstem neuromodulatory systems such as serotonergic systems. The DR, along with the MnR, is the major source for 5-HT in the brain (Steinbusch, 1981). Although the DR is a main source for 5-HT, it also contains neurons that express other neurotransmitters and neuropeptides, including CRF. Corticotropin-releasing factor-immunoreactive neurons have been observed in the DR in colchicine-treated rats (Commons et al., 2003). Corticotropin-releasing factor-positive neurons were predominately found in the dorsomedial subregion of the mid-rostrocaudal DRD with smaller numbers of positive cells in the ventrolateral part of the DRVL/VLPAG. Importantly, these CRF-immunoreative cells were mostly dual labeled for TPH indicating that these were also serotonergic neurons. In addition, CRF-positive cells were largely absent from the ventromedial and most caudal portions of the DRD and DRVL/VLPAG while CRF-positive fibers were seen to traverse the lateral edge of the rostral DRV. It was also demonstrated through anterograde tracing that the dorsomedial neurons had dense projections to the CE, a region involved in the control of emotion such as fear and anxiety (Gray, 1993; Davis, 1997; Commons et al., 2003; Phelps and LeDoux, 2005). Moreover, CRF application in the mid-rostrocaudal DRD increases 5-HT in the CE and freezing behaviors (Forster et al., 2006). Because the CE is involved in emotion and has direct CRF connections with specific regions of the DR, it is situated to control serotonergic systems and modulate emotion-related behavioral output.

The dorsomedial neurons of the mid-rostrocaudal DR have dense CRF projections to the BNST while the BNST also has reciprocal connections with the DRD and DRC (Van Bockstaele et al., 1993; Petit et al., 1995; Peyron et al., 1997; Dong et al., 2001; Commons et al., 2003; Dong and Swanson, 2004). The BNST has been shown to be an important region controlling emotional behaviors such as alterations in acoustic startle behavior (Davis et al., 2010). CRF innervation of the DRD/DRC by BNST CRF positive neurons has not been directly shown, however, control of emotional-like behavior by CRF projections from the BNST to the caudal DRD has been suggested as BNST lesions block escape deficiencies produced after inescapable shock while administration of CRF into the DRC mimics the effects of inescapable shock on inhibiting escape behaviors (Hammack et al., 2002, 2004). Moreover, overexpression (OE) of CRF in the BNST induces a decrease in CRF_2_ receptor mRNA specifically within the DRD suggesting that the BNST has direct CRF projections to this brain region (Sink et al., 2013). Given the role of the BNST in control of emotional behaviors and its potential connections with subregions of the DR, it is an important structure that most likely contributes to control of emotional behavioral output through CRF-5-HT interactions.

The distribution of CRF receptors within the DR is also topographically organized and both CRF_1_ and CRF_2_ receptors are colocalized with serotonergic neurons as well as non-serotonergic neurons (Day et al., 2004; Waselus et al., 2009). CRF_1_ receptor mRNA density is considerably lower than CRF_2_ receptor mRNA in the DR (Van Pett et al., 2000). CRF_1_ receptors, using immunohistochemistry and electron microscopy, are present on the plasma membrane of dendrites in the DR as well as within the cytoplasm in roughly equal distribution (Waselus et al., 2009). CRF_1_ receptors are located in the dorsal portion of the DRVL/VLPAG and have been shown to be colocalized there with GABA in 36% of neurons triple labeled for c-Fos, GABA, and CRF_1_ receptors after forced swim stress (Roche et al., 2003; Day et al., 2004). These data suggest that CRF receptor activation can serve to modulate serotonergic activity both directly and indirectly.

CRF_2_ receptors are topographically organized in the DR and are expressed in both serotonergic and non-serotonergic neurons. CRF_2_ receptors have been demonstrated using immunohistochemistry and electron microscopy in the DR on both axon terminals and in dendrites with a predominant level within the cytoplasm (Waselus et al., 2009). The CRF_2_ receptor is apparent in low numbers in the rostral portion of the DR but increases in greater number in more caudal sections of the DR as shown with *in situ* hybridization histochemistry and immunoreactivity (Day et al., 2004; Lukkes et al., 2011). In addition, the effects of CRF administration in the caudal portions of the DR is only blocked by CRF_2_ but not CRF_1_ receptor antagonists (Hammack et al., 2002, 2003a; Staub et al., 2005, 2006). Further, CRF_2_ receptors also show a topographically organized colocalization with 5-HT neurons and GABA neurons in the DR (Day et al., 2004). Specifically, CRF_2_ receptor mRNA in the rostral and mid-rostrocaudal levels of the DR is almost exclusively colocalized with 5-HT neurons while in more caudal regions, about half of the CRF_2_ receptor mRNA is colocalized in GABAergic neurons (Day et al., 2004).

CRF and UCN 1 fibers are topographically organized in the DR but much less is known about UCN 2 and UCN 3 fibers. Corticotropin-releasing factor-containing axons are more dense in the medial ventral portion of the rostral DR and then more dense in the dorsolateral DR in more caudal regions with less density in the medial ventral portion (Valentino et al., 2001). This topographical organization suggests that CRF is able to modulate 5-HT in a complex and dynamic manner to influence emotional behavior given that 5-HT neurons are also topographically organized (Hale and Lowry, 2011; Hale et al., 2012). UCN 1 fibers have also been described in the DR including within DRD, DRV, and DRVL subregions suggesting the possibility that UCN 1 also influences serotonergic systems and emotional behavior through CRF_2_ (Vaughan et al., 1995). The physiological involvement of UCN 2 and 3 in the modulation of the DR and 5-HT is little known as the DR shows very sparse or no innervation by either UCN 2 or 3 (Reyes et al., 2001; Li et al., 2002). Nevertheless, selective activation of CRF_2_ receptors in the DR does modulate and mediate emotional behaviors (described below), suggesting that further work looking into the role of the UCN's in the DR is necessary to elucidate their physiological role.

## Mechanisms of CRF/5-HT interactions

### CRF and UCNs effects on DR neuronal firing

CRF has been shown to alter DR neuronal firing rates *in vivo* and *in vitro*. Studies looking at the response of DR neurons *in vivo* have shown a bimodal response to CRF within the medial rostral portion of the DR in that low doses given either intracerebroventricularly (i.c.v) or directly into the DR inhibit neuronal firing while higher doses increase firing (Kirby et al., 2000; Price et al., 2002) consistent with other data that show a similar pattern of effects on 5-HT release in the lateral striatum and the lateral septum (Price et al., 1998; Price and Lucki, 2001). The response of 5-HT neurons in the DR to CRF has been shown to be topographically organized *in vitro* with more serotonergic neurons responding in the ventral portion of the DR compared to the dorsomedial aspect of the DR (Lowry et al., 2000). Although the majority of neurons in this region respond with an increase in firing rates, many were non-responsive and several showed a decreased firing rate. Stress has been shown to alter the response of serotonergic neuron firing rates after CRF application in that more neurons respond with increased firing rates and have a greater rate of firing compared to control animals, suggesting that CRF plays an important role after stress to modulate the serotonergic system (Lowry et al., 2000). Further studies have shown that CRF_2_ receptors move from being predominately within the cytoplasm and then move to the plasma membrane after stress (Waselus et al., 2009) although the mechanism for increased responsiveness and increased firing rates in serotonergic cells to CRF after stress is not known. These data thus show that the interaction between CRF and 5-HT on neuronal activity is modulated by the region of the DR, the amount of CRF released, and prior experience.

Further substantiating the evidence that the members of the CRF family of peptides can have multiple influences on 5-HT, CRF_2_ receptors seem to play a dual role in the DR on neuronal firing while CRF_1_ receptor activation seems to be inhibitory (Kirby et al., 2000). Small amounts (0.1 − 10 ng) of UCN 2 injected into the mid-rostrocaudal DR inhibit neuronal firing in 5-HT neurons whereas higher amounts (30 ng) of UCN 2 increase firing in 5-HT neurons and this effect is blocked by selective CRF_2_ receptor antagonists (Pernar et al., 2004). Furthermore, this high dose of UCN 2 also inhibited more non-5-HT neurons than the low dose which could indicate inhibition of GABA neurons through CRF_2_ receptors, which would disinhibit 5-HT neurons and result in increased serotonergic firing. CRF_1_ receptors also appear to be involved in the inhibition of neuronal firing after CRF administration in the DR as this effect can be blocked with a specific CRF_1_ receptor antagonist (Kirby et al., 2000).

### CRF and UCNs effects on serotonergic neurons as measured by c-Fos

The members of the CRF family of neuropeptides induce topographically organized neuronal activation as measured by c-Fos. CRF, when infused i.c.v., produces a topographically organized neuronal activation within the DR in medial prefrontal cortex-projecting neurons with a higher percent of activation in caudal portions of the DR as measured by c-Fos (Meloni et al., 2008). Specifically, i.c.v. infusion of CRF (1 μg), induces c-Fos positive neurons in the entire DR, with higher numbers of positive cells seen in more caudal regions compared to more rostral regions (Meloni et al., 2008). UCN 1 (1–10 μg) given i.c.v. has also been shown to increase c-Fos in the DR although topographical activation has not been described (Bittencourt and Sawchenko, 2000). Additional studies show topographically organized activation of serotonergic and non-serotonergic DR neurons by i.c.v. infusion of 2 μg of UCN 2 (Staub et al., 2005, 2006; Hale et al., 2010). Specifically, c-Fos was seen in the mid-rostrocaudal DRD and the DRC and this activation was blocked by a specific CRF_2_ receptor antagonist. Consistent with these data, direct infusion of UCN 2 (100 ng) into the DR also produces increased c-Fos within the DR but the effect is more widespread throughout the DR and again this is blocked using a specific CRF_2_ receptor antagonist (Amat et al., 2004). Moreover, UCN 2 activation of DR neurons includes activation of ventricle/periventricular-projecting serotonergic neurons as well as non-ventricle/periventricular-projecting serotonergic neurons, suggesting that CRF_2_ ligands could play an important physiological role in behavioral consequences of CRF_2_ receptor activation although further examination is needed (Amat et al., 2004; Hale et al., 2010). Taken together, these data show that both CRF_1_ and CRF_2_ receptor agonists activate DR neurons in a topographically organized fashion such that the mid-rostrocaudal and caudal portions of the DR appear to be preferentially activated, suggesting that these regions are important in CRF-5HT interactions.

### CRF receptor activation alters serotonergic neurotransmission as measured by microdialysis

CRF receptor activation following i.c.v. administration of CRF or CRF-related neuropeptides induces changes in extracellular 5-HT concentrations in specific brain regions involved in control of emotional behavior, including the hippocampus, as measured by microdialysis (Linthorst et al., 2002;; Kagamiishi et al., 2003; De Groote et al., 2005). However, chronic i.c.v. CRF infusion (1 μg/1 μL/h for 7 days) produces no basal difference in 5-HT levels in the hippocampus but does blunt the elevation in 5-HT after LPS injection, suggesting chronic elevation of CRF blunts stress-induced release of 5-HT in projection regions of the DR (Linthorst et al., 1997). While CRF receptor activation following i.c.v. administration of CRF or CRF-related neuropeptides can increase 5-HT release, it can also decrease release. I.c.v. CRF produces a decrease in 5-HT release in both the lateral septum and lateral striatum at a low dose (0.3 μg) while this manipulation either does not change 5-HT concentrations or increases concentrations at a higher dose (3.0 μg) (Price et al., 1998, 2002; Price and Lucki, 2001). Both 5-HT_1A_ and _2A_ receptor activation in the lateral septum has been associated with increased anxiety-like behavior, which would correspond with increased release of 5-HT in this region (Cheeta et al., 2000; de Paula et al., 2012). The decrease in 5-HT release in the lateral septum may allow for more proactive behaviors such as exploration to occur when placed in a mildly stressful situation (low dose of CRF) while a very stressful (high CRF dose) situation could induce more reactive behaviors like freezing. Because CRF receptors are located in other regions besides the DR, these effects of i.c.v CRF receptor agonists likely result from activating circuits that are connected with the DR as well as directly activating receptors within the DR.

Intra-DR CRF receptor activation induces changes in 5-HT concentrations in specific emotion-related brain regions, similar to effects seen with i.c.v. administration of CRF receptor agonists. CRF (0.5 μg) injected into the medial portion of the DR, including both the dorsal and ventral aspects, increases 5-HT release in the prefrontal cortex after a 60 min delay (Forster et al., 2006). Activation of the DR by 0.5 μg CRF injection also produces an immediate increase in 5-HT in the CE (Forster et al., 2006; Lukkes et al., 2008; Scholl et al., 2010). Further, CRF_2_ receptor activation in the caudal DR produces an increase in 5-HT in the BLA and increases c-Fos in serotonergic neurons within the rostral, mid-rostrocaudal, and caudal DR (Amat et al., 2004). Serotonin concentrations in the Acb are also modified by CRF receptor activation in the DR. Low doses of CRF (0.1 ug) injected into the DR, including both the DRD and DRV aspects of the mid-rostrocaudal DR, reduces 5-HT concentrations whereas higher doses (0.5 μg) increases 5-HT in the Acb (Lukkes et al., 2008). Importantly, a CRF_1_ receptor antagonist blocked the effect at the low dose while a CRF_2_ receptor antagonist blocked the effect of the high dose, showing that CRF is activating CRF_1_, perhaps on GABA interneurons, to inhibit 5-HT release while at higher concentrations is also activating CRF_2_ receptors, resulting in increased release of 5-HT in the Acb. These data show that direct activation of CRF receptors in the DR modulate 5-HT release in emotion-related brain regions.

The changes in 5-HT release in the BLA, CE, and Acb are associated with varying behavioral outputs related to emotion. Activation of 5-HT_2C_ receptors in the BLA increases fear-like behaviors (Campbell and Merchant, 2003; Greenwood et al., 2012) which corresponds with the increased release of 5-HT seen in this brain region after CRF_2_ activation in the DR (Amat et al., 2004). Additionally, 5-HT receptor activation in the CE has also been implicated in increased anxiety and fear-like behaviors as increased 5-HT in the CE leads to increased freezing behavior (Forster et al., 2006), presumably through activation of the excitatory 5-HT_2A/C_ receptors located in the CE (Asan et al., 2013). Further, foot shock has been shown to also increase 5-HT release in the Acb in association with freezing behavior, indicating a role for fear-like behavioral responses in relation to increased 5-HT release in the Acb (Fulford and Marsden, 1997, 2007). It is not clear which 5-HT receptors in the Acb would be playing a role in fear-like behaviors. However, both 5-HT_2A_ and5-HT_2C_ receptors in this region have been shown to be involved in drug reward behavior, which can be modulated by stress (Erb and Stewart, 1999; Zayara et al., 2011). It is likely that increasing and decreasing 5-HT levels in various emotion-related brain regions involves a complex interplay of 5-HT receptors given that their binding can both inhibit and excite neuronal activation and 5-HT receptors have been shown on GABA neurons and glutamate neurons (Rainnie, 1999; Guo and Rainnie, 2010; Asan et al., 2013).

## CRF, UCNs, and 5-HT interactions controlling emotional behavior

Numerous studies have demonstrated the involvement of members of the CRF family of peptides and their respective receptors and 5-HT in emotional behaviors in rodents. These include studies involving administration of specific CRF receptor agonists and antagonists with both i.c.v. and intra-DR applications as discussed above. Additionally, development of numerous mutant mice with genetic knock out (KO) or OE of one or more of these peptides or receptors have helped further our knowledge about the important role CRF plays in emotional behaviors (described below). Here, we will focus on behaviors that have been shown to involve the interactions between CRF, the UCNs, and 5-HT.

A number of studies have implicated CRF/5-HT interactions in control of emotional behavior. Administration of CRF through i.c.v increases the acoustic startle response and the CRF-induced startle is correlated with activation of c-Fos within the DR (Meloni et al., 2008). Partially, the c-Fos positive neurons were also positive for 5-HT and projected to the mPFC, a region that is important in the effects of controllability on behavioral consequences of stress (Rozeske et al., 2011; Patel et al., 2012). Behavioral consequences of uncontrollable stress are mediated by CRF receptors in the DR (Hammack et al., 2002, 2003a,b). In particular, it has been shown that CRF_2_ receptors in the DRC are responsible for the behavioral consequences, observed 24 h later, of inescapable shock and that activation of CRF_2_ receptors in the DRC can mimic the effects of uncontrollable shock on behavior (Hammack et al., 2002). Further, activation of CRF_1_ receptors by low doses of CRF acts to inhibit the DR and can block the behavioral consequences of uncontrollable stress (Hammack et al., 2003a). Stressors such as foot shock and restraint induce neuronal activation, as measured by c-Fos, in anxiety-related regions including the DR, amygdala, and BNST while at the same time also increasing mRNA for CRF in the BNST and CE, suggesting that anxiety- or fear-inducing stimuli alter CRF function while activating neurons in the DR (Funk et al., 2006). Importantly, this implicates a complex system that is responsive to the effects of an acute stressor, which can lead to alterations in emotional behaviors such as increased freezing behaviors (Hammack et al., 2004). These data thus suggest a crucial role of CRF receptors within the DR in control of stress-related behaviors and suggest that CRF/5-HT interactions are important in the behavioral consequences of uncontrollable stress.

Chronic activation of the CRF system is associated with changes in emotional behavior. Specifically, OE of CRF within the BNST does not produce basal changes in anxiety yet when induced prior to fear conditioning, it interferes with learning, while induction after fear conditioning but before fear testing produces an exaggerated fear response (Sink et al., 2013). Importantly, OE of CRF in the BNST results in changes in binding density for CRF_1_ in the BNST and CRF_2_ in the dorsal and caudal portions of the DR. This suggests that alterations of CRF expression in specific areas connected to the DR can lead to both physiological and emotional changes, depending on time points, and can disrupt fear learning or enhance fear expression, possibly through alterations in 5-HT function through decreased CRF_2_ receptors in the dorsal and caudal DR as these receptors have been shown to be involved in fear related behaviors (Hammack et al., 2003a). Repeated administration of UCN 1 into the BLA has also been shown to induce serotonergic changes in the DR, including an increase in *tph2* mRNA in specifically the DRVL that was correlated with increases in anxiety-like behavior (Donner et al., 2012a). Further, maternal separation and later social defeat also serve to increase *tph2* mRNA in the DRVL/VLPAG and produce a more passive-like coping behavior, and this increase in *tph2* mRNA could serve as a common factor related to altered emotionality brought on by multiple environmental stressors (Gardner et al., 2005, 2009). These studies suggest that the interaction between CRF receptor activation and 5-HT can modify emotional behavior while environmental experience can serve to alter 5-HT systems in a manner that is dependent on CRF receptor activation.

Interactions between CRF and serotonergic systems have also been implicated in control of active vs. passive behavioral coping responses during forced swim stress. The swim stress-induced reduction in 5-HT concentration in the lateral septum has been shown to be dependent on CRF receptor activation as an i.c.v. CRF_1,2_ receptor antagonist blocks this effect on 5-HT (Price et al., 2002). This effect may be specific to swim stress, however, as 5-HT concentrations increase in the lateral septum in mice in the presence of predator odor (Beekman et al., 2005). Further, the increase in 5-HT seen in the hippocampus during forced swim can also be blocked using a non-specific CRF antagonist given i.c.v. (Linthorst et al., 2002;; Kagamiishi et al., 2003; De Groote et al., 2005). These studies suggest a role for CRF receptor activation in control of serotonergic systems by diverse stress-related stimuli.

## CRF, UCN 1, 2, and 3, and CRF receptor transgenic animals, serotonergic systems, and emotional behavior

One line of research taken to investigate the roles of CRF, UCNs, and CRF receptors in control of serotonergic systems and emotional behavior is to use transgenic animals. Genes can be removed or added in to influence development from fertilization or can be conditionally changed after birth to avoid developmental alterations associated with transgenic manipulations that may lead to unintended consequences (Smith et al., 1998; Timpl et al., 1998). It is important to keep in mind as well-that many of these studies will produce essentially the overall sum effect of adding or removing peptides or receptors from the entire brain and peripheral systems on behavior and that further work with more selective changes will be informative as to their roles within specific brain regions.

Both CRF_1_ and CRF_2_ receptor KO mice have been developed and used to investigate the role of the receptors in control of emotional behaviors. The CRF_1_ receptor KO mice display a decrease in anxiety-like behaviors while CRF_2_ receptor KO mice tend to display an increase in anxiety and depression-like behaviors, although not in all cases or in all measures (Smith et al., 1998; Timpl et al., 1998; Bale et al., 2000; Coste et al., 2000, 2006). In CRF_1,2_ receptor double KO mice, only males show an increase in anxiety-like behavior while the females tend to show normal or decreased anxiety, which suggests that there is an interaction between sex and genotype on anxiety-like behavioral output in these animals (Bale et al., 2002). The rearing behavior of heterozygous and homozygous CRF_2_ receptor KO dams seems supports an increase in anxiety-like behavior in their male offspring regardless of the males' genotype, implying that both environmental and genetic factors play a role in anxiety-like behaviors and that sex is an important factor (Bale et al., 2002). Further evidence suggests that CRF_1_ receptors in the limbic system specifically are important in controlling anxiety-like behaviors (Muller et al., 2003). Conditional KO of CRF_1_ receptors in the limbic system has no effect on the HPA axis, but results in reduced anxiety-like behavior and increased active coping in depression models (Muller et al., 2003). CRF_1_ receptors in the limbic system therefore seem to play a critical role in initiating an anxiety response.

Mouse models have also been developed to investigate the effects of OE or deletion of CRF. Chronic OE of CRF results in a downregulation of UCN 1 in the Edinger-Westphal nucleus (Kozicz et al., 2004) while CRF KO results in an upregulation of UCN 1 in the Edinger-Westphal nucleus, suggesting that CRF may control the level of UCN 1 expression or that changes in CRF can be compensated for by UCN 1 (Weninger et al., 2000). Moreover, CRF OE mice also show a change in CRF_1_ and CRF_2_ receptor mRNA expression throughout the brain (Korosi et al., 2006). In particular, OE of CRF induces a down-regulation of CRF_1_ receptors while at the same time it induces an upregulation of CRF_2_ receptors in the brain while the overall distribution of receptors remains the same showing that receptor mRNA expression is dependent on the level of CRF expression in these animals. CRF OE mice also show an increase in anxiety-like behavior, perhaps mediated in part by overactivation of CRF_1_ receptors or by the increase in CRF_2_ receptors in the DR (Korosi et al., 2006) and changes in responsiveness to alterations in 5-HT release in regions connected with the DR (Stenzel-Poore et al., 1994; Heinrichs et al., 1997; van Gaalen et al., 2002). These mouse models demonstrate that CRF KO or OE alters the expression of other members of the CRF family of neuropeptides and their receptors, implying that an overall increase or decrease of CRF or the UCNs can contribute to changes in receptor expression and alter behavioral output.

Chronic OE of UCN 3 is associated with changes in the serotonergic system and altered emotion-like behaviors. Chronic OE of UCN 3 produces a change in post-stress 5-HT and 5-HIAA concentrations in the caudal and dorsal DR and lateral septum as well as a basal change in 5-HT_1A_ receptor mRNA in both the DR and amygdala (Neufeld-Cohen et al., 2012). UCN 3 OE also alters basal anxiety-related behavior compared to wild type animals (Neufeld-Cohen et al., 2012), which may implicate desensitized CRF_2_ receptors resulting in the increased anxiety, similar to the CRF_2_ receptor KO mice. In particular, UCN 3 OE mice have increased anxiety-like behavior in both the elevated plus-maze and the light-dark box and an increase in immobility in the tail suspension test suggesting that chronic CRF_2_ receptor activation results in a basal increase in emotionality. Consistent with these data, conditional OE of UCN 3 in the rostral portion of the perifornical area, where UCN 3 is normally expressed, also increases anxiety-like behavior (Kuperman et al., 2010). Interestingly, post-stress anxiety-like behavior in UCN 3 OE mice is either not increased or is in fact reduced suggesting that chronic activation of CRF_2_ receptors creates a chronic anxiety-like state but resistance to further stress-induced anxiety (Neufeld-Cohen et al., 2012).

A number of studies have also investigated the role of UCN 1, 2, and 3 in emotional behaviors using transgenic mice. UCN 1 KO mice have been shown to have normal anxiety-like behavior although a decrease in the acoustic startle response is seen in males (Wang et al., 2002) and a lower startle response has been associated with decreased anxiety in other measures of anxiety (Salam et al., 2009). These animals are described as having normal hearing although another line of UCN 1 KO mice appeared to have disruption of hearing and an increase in anxiety-like behavior (Vetter et al., 2002) so a reduced startle response maybe due do a disruption in the startle neuronal pathway. Both UCN 2 and 3 KO animals do not show any disruption in anxiety-like behaviors, although reduced aggressiveness is seen in male UCN 2 KO's and reduced depression-like behaviors were noted in female UCN 2 KO mice (Chen et al., 2006; Deussing et al., 2010; Breu et al., 2012). However, given that both CRF and UCN's can activate CRF_2_ receptors, it is not surprising that there is not a noticeable change in anxiety or consistent decrease in depression-like behaviors in the UCN 2 or 3 KO mice. A double KO of UCN 1 and 2, however, results in a anxiolytic-like phenotype (Neufeld-Cohen et al., 2010a) while UCN triple KO mice show normal basal anxiety levels but increased anxiety-like behavior 24 h after a stressor (Neufeld-Cohen et al., 2010b). Notably, the UCN triple KO mice show some basal and 24 h post-stress differences in serotonergic activities in the amygdala, subiculum and medial and later septum, which is consistent with the hypothesis that the increase in anxiety-like behavior is mediated in part by changes in serotonin and implicates a role for CRF_2_ receptor activation in controlling 5-HT and its potential role during the stress recovery period.

## Non-human primate data on CRF/5-HT interactions and emotional behavior

Non-human primate data gives further insight into the interaction between CRF, serotonergic systems, and emotional behavior. Recent data suggest that, in a particular subset of cynomolgus macaques deemed to be more stress-sensitive than their cohorts because of interrupted menstrual cycles, chronic treatment with a serotonin selective reuptake inhibitor (SSRI) produces significant changes in CRF receptors after 15-weeks of administration but not in less-stress sensitive animals (Senashova et al., 2012). Specifically, after chronic treatment with an SSRI, the stress sensitive monkeys had an increased number of CRF_2_ receptor mRNA positive cells as found through digoxigenin-*in situ hybridization* staining, compared to less stress-sensitive monkeys. Similar studies have shown that the stress-sensitive monkeys have an increased cortisol response to serotonin release induced by fenfluramine while showing a blunted prolactin release (Bethea et al., 2005a) and basal increased cortisol release during the day (Herod et al., 2011a,b), suggesting further disruption in normal serotonin systems. Interestingly, these monkeys, along with the altered CRF receptors in response to an SSRI, also have lowered CRF fiber density in the DR with an increased number of UCN 1 cell bodies after SSRI treatment (Weissheimer et al., 2010). This suggests that altered levels of CRF and UCN 1 contribute to the stress sensitivity witnessed in the animals and that altered 5-HT systems contribute to these differences, perhaps through increased CRF or decreased UCN signaling to the DR. In fact, stress-sensitive cynomolgus macaques, compared to less stress sensitive cynomolgus macaques, have decreased serotonin transporter and tryptophan hydroxylase 2 (TPH) mRNA and, in one study, had lower 5-HT_1A_ receptor mRNA in the DR, all suggesting that alterations in these 5-HT systems contribute along with the altered CRF/UCN system to the stress-sensitive phenotype of these monkeys (Bethea et al., 2005b; Lima et al., 2009).

## Clinical data on CRF/5-HT interactions and emotional behavior

There is a wealth of knowledge gained from studies showing that SSRI's can be a useful way to treat anxiety and depression (Goldstein and Goodnick, 1998) although a meta-analysis of clinical data also show that this may be dependent on the severity of the disorder and less useful in mild to moderate depression (Fournier et al., 2010) although see (Gibbons et al., 2012). There has also been interest in modulating CRF in patients with affective disorders to help alleviate their symptoms (Künzel et al., 2003). Corticotropin-releasing factor-positive terminals are apposed to serotonergic neurons in the human brain suggesting that they play a role in modulating 5-HT as seen in animal models (Ruggiero et al., 1999). Importantly, elevated CRF concentrations have been described in the cerebral spinal fluid in suicide victims, suggesting a dysregulation of the central CRF system (Arató et al., 1989). Although the data have not been completely consistent in findings of elevated CRF concentrations, with reports of no difference or even decreased CRF in CSF, it does appear that there can be abnormal levels of CRF in CSF in people with depression and the discrepancies may represent subgroups of depression or perhaps how long they have been in the episode of depression (Mitchell, 1998). One study of interest concerning the interplay between CRF and 5-HT showed elevated levels of CRF in CSF which subsequently normalized after treatment with the SSRI fluoxetine, suggesting that the abnormality in 5-HT function in depression could be related to the elevation of CRF (De Bellis et al., 1993).

Depression has also been associated with alterations in 5-HT function in specific regions of the DR in humans. Data collected from postmortem human brain tissue show an increase in TPH immunoreactivity (Underwood et al., 1999; Boldrini et al., 2005) and mRNA (Bach-Mizrachi et al., 2006, 2008) in the DR of depressed suicides, with a more pronounced increase in the DRC. Data also show an increase of TPH immunoreactivity specifically in the DRD of depressed alcoholic suicides (Bonkale et al., 2006) with a trend for an increase in *tph2* mRNA expression in the same region in non-alcoholic depressed suicides (Bach-Mizrachi et al., 2006). These data are in contrast to those described in the stress-sensitive monkeys in which it had been found that there is decreased *tph2* mRNA compared to less stress sensitive monkeys, which may indicate that there are multiple pathways leading to emotion-related disorders (Bethea et al., 2011). In male, but not female, suicide victims, *UCN* mRNA is significantly elevated in the Edinger-Westphal nucleus (Kozicz et al., 2008) further implicating a disruption in central CRF-related peptides related to depression. Other data show that CRF is upregulated as well in the DR and MnR in depressed suicides providing direct evidence for changes in CRF in brain regions that are the major sources for 5-HT in humans, which could be one reason for the altered TPH seen in other studies, perhaps through increased activation of serotonergic neurons by CRF (Austin et al., 2003).

## Conclusions

Corticotropin releasing factor and the UCNs interact with serotonergic systems in a topographically organized manner and, depending on the receptor and the connectivity with limbic brain regions and concentrations of peptide, can lead to alterations in gene expression, changes in serotonergic output, and increased or decreased emotional behaviors. Focus on the relationship between the members of the CRF family of peptides and serotonergic systems should take into consideration the complex topographical organization of serotonergic systems. Increased understanding of these relationships in specific brain regions could lead to novel therapeutic strategies to more directly modulate emotional outcomes with fewer side effects relative to current treatments for anxiety and affective disorders.

### Conflict of interest statement

The authors declare that the research was conducted in the absence of any commercial or financial relationships that could be construed as a potential conflict of interest.
